# Reduced volume of diabetic pancreatic islets in rodents detected by synchrotron X-ray phase-contrast microtomography and deep learning network

**DOI:** 10.1016/j.heliyon.2023.e13081

**Published:** 2023-01-19

**Authors:** Qingqing Guo, Abdulla AlKendi, Xiaoping Jiang, Alberto Mittone, Linbo Wang, Emanuel Larsson, Alberto Bravin, Erik Renström, Xianyong Fang, Enming Zhang

**Affiliations:** a School of Computer Science and Technology, Anhui University, Hefei, China; b Islet Pathophysiology, Department of Clinical Science, Lund University Diabetes Centre, Malmö, Sweden; c School of Physical Science and Technology, Southwest University, Chongqing, China; d Advanced Photon Source, Argonne National Laboratory, Lemont, IL, United States; e Biomedical Beamline ID17, European Synchrotron Radiation Facility, Grenoble Cedex, France; f Division of Solid Mechanics & LUNARC, Department of Construction Sciences, Lund University, Lund, Sweden; g NanoLund, Lund University, Box 118, 22100, Lund, Sweden; h Department of Physics, University Milano Bicocca, Milan, Italy; i Department of Physics, Università della Calabria, Rende, Italy

**Keywords:** Diabetes, Pancreatic islets, X-ray microtomography, Deep learning, Synchrotron radiation, X-ray phase-contrast, SRμCT, synchrotron radiation X-ray phase-contrast microtomography, T1D, type-1 diabetes, T2D, type-2 diabetes, PCI, phase contrast imaging, AAM, affinity-aware module, SF, Shortcut-Free, AA-Net, Affinity-Aware Network, STZ, streptozotocin

## Abstract

The pancreatic islet is a highly structured micro-organ that produces insulin in response to rising blood glucose. Here we develop a label-free and automatic imaging approach to visualize the islets *in situ* in diabetic rodents by the synchrotron radiation X-ray phase-contrast microtomography (SRμCT) at the ID17 station of the European Synchrotron Radiation Facility. The large-size images (3.2 mm × 15.97 mm) were acquired in the pancreas in STZ-treated mice and diabetic GK rats. Each pancreas was dissected by 3000 reconstructed images. The image datasets were further analysed by a self-developed deep learning method, AA-Net. All islets in the pancreas were segmented and visualized by the three-dimension (3D) reconstruction. After quantifying the volumes of the islets, we found that the number of larger islets (=>1500 μm^3^) was reduced by 2-fold (wt 1004 ± 94 vs GK 419 ± 122, P < 0.001) in chronically developed diabetic GK rat, while in STZ-treated diabetic mouse the large islets were decreased by half (189 ± 33 vs 90 ± 29, P < 0.001) compared to the untreated mice. Our study provides a label-free tool for detecting and quantifying pancreatic islets *in situ*. It implies the possibility of monitoring the state of pancreatic islets in vivo diabetes without labelling.

## 1 Introduction

More than 400 million diabetic patients worldwide suffer from chronic hyperglycaemia and severe complications [1]. Patients with type-1 diabetes (T1D) and a subgroup of type-2 diabetes (T2D) show a severe reduction in pancreatic beta-cell mass [1]. In T1D, the beta-cell mass decreased to 5–20%, while in T2D, the beta-cell mass reduction is highly related to disease development. In most early-stage diabetes, although the patients were commonly undiagnosed, the damage of the beta-cell mass or islets of Langerhans has already occurred. This damage can consequently cause the alternation of the number and size of the islets. Notably, these alternations on islets are not always correlated with the changes in beta-cell mass. For example, a recent report showed that there were still a large number of beta cells in T1D, while the islets were utterly destroyed. Moreover, beta-cell *trans*-differentiation to alpha- or delta-cells during diabetes development might also alter the islet morphology [2,3]. Therefore, a non-bias imaging method to quantify islets in the pancreas is necessary for understanding the pathophysiology during disease development and for diagnosing early-stage diabetes.

Currently, the methods used to detect the islets, either in the sections of autopsy or in vivo animals, are based on the dye labelling of the beta-cells. Although the labelling relied on dyes such as fluorescent probes [4] or radiotracers (Positron Emission Tomography) [5] specifies the targeting, it also inevitably induces flaws that diminish the reliability of the detection, for example, 1) the dyes target only beta cells but not the entire islets; 2) the unspecific binding in the islet cells, particularly in vivo conditions, may cause various morphologic changes; 3) the conventional beams are not capable of reaching in the deeper tissue, where the dyes are often with the low dose. Therefore, developing a label-free and high-penetrating beamline imaging method can pave new ways for islet imaging. It will provide a deeper insight into the pathophysiology of diabetes, especially the progression of diabetes.

Hard X-ray-based imaging, such as SRμCT, has exhibited a powerful penetrating ability to distinguish the nuance of soft tissue architecture [6]. In combination with in-line propagation-based phase-contrast imaging (PCI) [7], it has shown prominent advantages in the imaging of the breast [8], liver [9] and central nervous system [10]; for example, the low X-ray dose imaging, the higher sensitivity with signal/noise ratio and revealing the distinct structure in the deep tissue inaccessible to conventional methods [11]. These advantages tempted us to apply the protocol for imaging the islets in the entire pancreas.

Deep learning brings remarkable advantages of image analysis and benefits dominantly in diagnosing related medical applications. Among the applications, global-oriented methods take advantage of weighted fusion to build feature abstraction, providing a high potential for soft tissue analysis [12,13]. However, these methods disregard the structure's similarities and thus are weak at inter-class discrimination and intra-class proximity. While the other methods may enhance the features of edges of soft tissues by inserting repeated blocks with encoder-decoder architectures, redundancy is unavoidable during this process, which may degrade the romance. To tackle these limitations, here we developed a novel encoder-decoder style neural model Affinity-Aware Network (AA-Net). By using the ID17 beamline at the ESRF (European Synchrotron Radiation Facility), Grenoble, France, we carried out the SRμCT imaging in the pancreas without labelling. and obtained the distinct structures of islets in the pancreas. Furthermore, using the self-developed AA-Net, we have, for the first time, accomplished imaging of all-natural islets in a pancreas.

## 2 Materials and methods

### 2.1 Animals and sample preparation

All the animal experiments conducted in this study were approved by the ethical committees at Lund University, Sweden (permission number 5.8.18–07202/2019 for mice and M87-14 for rats). Four C57BL/6J mice (Janvier Labs) were injected daily with 200 mg/kg streptozotocin in parallel four mice were daily injected with vehicle intraperitoneally for 5 consecutive days [14]. After 5 days from the injection, the mice were sacrificed. Four wild-type Wistar and four GK diabetic rats were purchased from Janvier Labs and sacrificed after the GK rats developed a diabetic state. The blood glucose of the mice and rats was monitored during the experiments. The blood glucose of STZ-treated mice raised more than 20 mmol/ml, which is considered a diabetic state. Then the mice were sacrificed and the pancreases were isolated surgically from the animals and placed in 0.8 ml 4% PFA in a 1.5-ml Eppendorf tube.

### 2.2 SRμCT

The experiment was performed at the ID17 station of the ESRF. The sample was mounted on a 5-axis tomography stage. The detection system was composed of a FReLoN CCD camera coupled with an indirect detection optic system [15] leading to a final pixel size of the acquired images of 7.8 × 7.8 μm^2^. During the acquisitions, the intact pancreas samples were inserted in a plastic tube filled with 4% PFA. Images were taken using the following settings: 1) continuous rotation mode at a speed of 0.3°/second; the total rotation range was 180°; the integration time of a single projection of 0.1 s; The X-ray beam was monochromatic with an energy of 30 keV. Image pre-processing, phase-retrieval and tomographic reconstructions have been performed using the PyHST2 software [16]. The reconstructed and analysed volumes present a voxel size of 7.8 × 7.8 × 7.8 μm^3^.

### 2.3 Confocal imaging

The islet structure in the pancreas was identified by confocal imaging and immunohistochemistry. The experiments were carried out following the recently published protocol [17]. Briefly, the entire pancreas was isolated, paraffin-embedded, cut into 5 μm sections and rehydrated for staining with primary antibodies of guinea pig anti-insulin (Eurodiagnostica), rabbit monoclonal anti-glucagon (Abcam). The secondary antibodies, donkey anti-guinea pig with conjugated cy2 (Jackson Immuno-lab), donkey anti-rabbit with conjugated cy5 (Jackson Immuno-lab) and Hoechst 33258 (Life Technologies), were used to detect insulin, glucagon, and nuclei, respectively. The images were acquired by confocal microscope (Meta 510, Zeiss, Germany) and proceeded by ZEN 2012 software.

### 2.4 Data pre-processing and augmentation

The resulting dataset consists of 20 volumes for two types of mice (diabetic and healthy), with each tomographic image slice size of 2048 × 2048 pixels. The image contrast between islets and the surrounding tissues is comparably low. Therefore, pre-processing is applied to obtain high-quality inputs. In particular, contrast limited adaptive histogram equalization (CLAHE) [18] is used to enhance the image contrast.

The ground truths of 49 images were manually labelled by experts in islet biology and then randomly divided into training (30 images) and testing (the rest images) sets. Each training image is cropped into overlapped 128 × 128 patches as inputs, while each testing one is cropped into 16 × 16 non-overlapped 128 × 128 patches for the final prediction after piecing their results.

The proposed model is conducted with PyTorch and trained with Adam [19] on a single GeForce GTX 1080 graphic card. The initial learning rate is 1e-5, and the overfitting is reduced by adopting an L2 regularization (where the weight decay is 0.01). The batch size for training is 16, with the maximum epochs set to 500.

### 2.5 Affinity-aware module (AAM)

Long-range dependencies are essential in deep-learning networks [20,21]. Based on these reports, this module computes the weights for each pixel regardless of their positions in the spatial dimension. Therefore, appearance similarities of pixels from the whole image are utilized for augmenting the correlations of distant pixels and thus obtaining the best object abstraction. The feature of a pixel at a specific position is influenced by all the pixels of the feature maps, not just by its neighbors. This non-local attention mechanism was used to measure the spatial correlation and contribute to creating effective global contexts.

The non-local relationship among distant pixels was assessed by the correlation between pixel features, which was performed through affinity computation. Formally, let $H\in {\mathbb{R}}^{C\times H\times W}$ be the feature map output by the encoder, with *C*, *H* and *W* being the number of input channels, the height and width of the feature map, respectively. We first convolute ***H*** with two groups of 1 × 1 kernel filters and flatten all feature maps to produce ***Q*** and ***K***, where $\left\{Q,K\right\}\in {\mathbb{R}}^{C^{'}\times N}$. Here, $C^{\prime }$ represents the reduced channel dimension with *N* = *H* × *W*. Then, the pixel-wise feature affinity among all pixels can be computed by:

(1) $$W=Q^{T}K$$

with $W\in {\mathbb{R}}^{N\times N}$ in Equation (1) indicating the degrees of correlations among all pixel features. W is then normalized by the *softmax* function in the row dimension as ***A:***

(2) $$A={\left(diag\left(\exp\left(Q^{T}K\right)•1\right)\right)}^{-1}\;\exp\left(Q^{T}K\right)$$

where **1** in Equation (2) is the column vector whose elements are all 1.

Similar features should promote each other with the dissimilar ones suppressed for a long-range oriented feature abstraction. This can be fulfilled by the normalized affinity matrix **A,** which can act as weights applied to feature ***H***. Here, the channel number of *H* is reduced by the 1 × 1 convolution such that $V\in {\mathbb{R}}^{C^{'}\times H\times W}$ is reshaped into $V^{'}\in {\mathbb{R}}^{C^{'}\times N}$. Consequently, matrix multiplication is performed between $V^{'}$ and ***A*** to obtain a weighted feature $H^{'}$, as formulated in Equation (3):

(3) $$H^{'}=AV^{'}$$

At last, $H^{'}$ is reshaped to which is then concatenated with ***H*** so that the final abstracted feature ***P*** can be obtained by restoring its original size through 1 × 1 convolution,

(4) $$P=f_{v}\left(f_{c}\left(H,H^{''}\right)\right)\text{.}$$

*f*_*v*_ and *f*_*c*_ in Equation (4) represent the 1 × 1 convolution and concatenation operations, respectively.

### 2.6 The twin-block based encoder

The encoder takes advantage of the popular ResNet block for multi-scale contexts and avoids gradient vanishing [22]. It also adopts the SF block, which is the reduced form of the ResNet block without the shortcut connection, to eliminate redundant information. Both ResNet and SF block perform 3 × 3 convolution, followed by batch normalization and ReLU activation. These operations are performed twice to enlarge the receptive field. This twin-block design combines multiple scales with less redundancy for efficient feature extraction, considering the simple structure of islets in the islet images.

Taking the twin blocks into the encoder and considering the multi-scale and conciseness requirements from feature extraction, we can design the encoder as follows. The SF block is positioned at the beginning and end of the encoder, while the ResNet block is performed four times in the middle of the encoder. Then, a six-block encoder is eventually obtained.

Given an input feature ***I*,** an SF block first extracts shallow features, which are then set as an input to four ResNet blocks for multi-scale extraction, with each ResNet block followed by a max-pooling layer to increase the receptive field. Then, an SF block extracts the abstract semantic features as the final encoder output. The gradient vanishing is removed through the shortcut connections of ResNet blocks, while the redundancy is eliminated by the SF blocks. This encoding process to obtain the output F can be formulated as in Equation (5):

(5) $$F=f_{d}{(f}_{r}^{4}\;(f_{d}\;(I\;)))$$

where *f*_*d*_ and *f*_*r*_ represent the operations of the ResNet and SF block respectively with $f_{r}^{4}$ denoting *f*_*r*_ applied four times.

### 2.7 AA-Net encoder-decoder structure

The deep learning framework AA-Net (Fig. S4A) takes the encoder-decoder structure. It consists of three parts: the encoder, AAM and the decoder. Firstly, the encoder is used to extract the features, where the twin-block based encoder is adopted to improve feature abstraction performance. AAM then enhances the abstraction by computing the spatial dependence for all pixels in the full domain with the appearance affinity operation. Finally, the decoder restores the high-level semantic features extracted from AAM for the image segmentation mask. The decoder is applied in the same way as the typical U-Net [23].

### 2.8 Loss function

The most common loss function is the cross-entropy loss function which can measure the similarities between the predicted values and their ground truths [24]. However, the distribution of islet pixels and background pixels is extremely imbalanced, i. e., the islet pixels are much fewer than the background ones in an islet image. Therefore, the binary cross-entropy can make the model prone to positive samples, decreasing its generalization ability. Therefore, a weighted binary cross-entropy is adopted to balance the contributions from both islet pixels and background ones:

(6) $$L_{w}=\sum_{i}\alpha \left(-t_{i}\;\log\;p_{i}\right)-\left(1-\alpha \right)\left(1-t_{i}\right)\log\left(1-p_{i}\right)$$

$\alpha =\left|t_{-}\right|/\left|t_{+}\right|+\left|t_{-}\right|$ where α in Equation (6) is defined as:

with |*t*_−_| and |*t*_+_| indicating the numbers of background and islets pixels, respectively.

A regularization loss *L*_*r*_ is also adopted to avoid overfitting [25]. Therefore, the final loss function is defined by Equation (7):

(7) $$L=L_{w}+L_{r}$$

### 2.9 Evaluation metrics of AA-Net processing on pancreas images

Several popular metrics are adopted to evaluate the performances, including sensitivity (SE), positive predictive value (PPV), Intersection Over Union (IOU) and the area under the Precision-Recall curve (AUPR). These metrics are formulated as Equation (8), Equation (9) and Equation (10), respectively.

(8) $$SE=\frac{TP}{TP+FN}$$

(9) $$PPV=\frac{TP}{TP+FP}$$

(10) $$IOU=\frac{A\cap B}{A\cup B}$$

where: 1) TP, TN, FP and FN represent the numbers of true positives, true negatives, false positives and false negatives, respectively; and 2) A and B represent pixel sets of the ground truths and their detection results, respectively. F-score is also important to measure the ratio of pixels predicted positive. Consequently, the larger the F-score, the better the model performance. Therefore, F-score defined in Equation (11) is adopted to choose the optimal model parameters during the training process.

(11) $$F-score=\frac{2\times SE\times PPV}{SE+PPV}$$

### 2.10 Certainty analysis

Certainty analysis is based on the certainty estimation of each pixel. The certainty of the *i-*th pixel, *c*_*i*__*,*_ is estimated as the proportion of the islet probability of this pixel to the max islet probability of all pixels in the same image: $c_{i}=\frac{p_{i}}{\max_{j\in islet}\left(p_{j}\right)}$, where p_*i*_, indicates the probability.

### 2.11 Islets segmentation and visualization

The reconstruction is based on the 2D segmentation masks computed by AA-Net, where 50 consecutive masks were obtained for each model (Wistar, GK, Control and 5-day STZ treatment). First, the islets are shown in different colors according to segmentation masks. Then the colored masks are input into ImageVis3D to visualize the 3D shapes of the islets.

### 2.12 Islet 3-D volumes quantification

The islet volume is computed based on the 2D segmentation slices. It is observed that the centers of the same islets from neighboring slices are offset. According to the experiment, the max offset γ is set 5, so that the islets from neighboring slices belong to the same islet if the distance of their centers is less than 5 pixels. The thickness of each islet in each slice is 7.8 μm with each pixel representing an area of 7.8 μm × 7.8 μm according to our experimental setup. The volume of an islet is calculated accordingly to its number of voxels.

### 2.13 Statistical analysis

The results for quantitative analysis are expressed as means ± SEM (Standard Error of the Mean) for the indicated number of repeats. The significance of random differences was analysed by Student's t-test or one-way ANOVA test. P value < 0.05 was considered a significant difference. All data were assessed to ensure normal distribution and equal variance among different groups.

## 3 Results

### 3.1 SRμCT imaging in the label-free pancreas of rodents

**Fig. 1:**
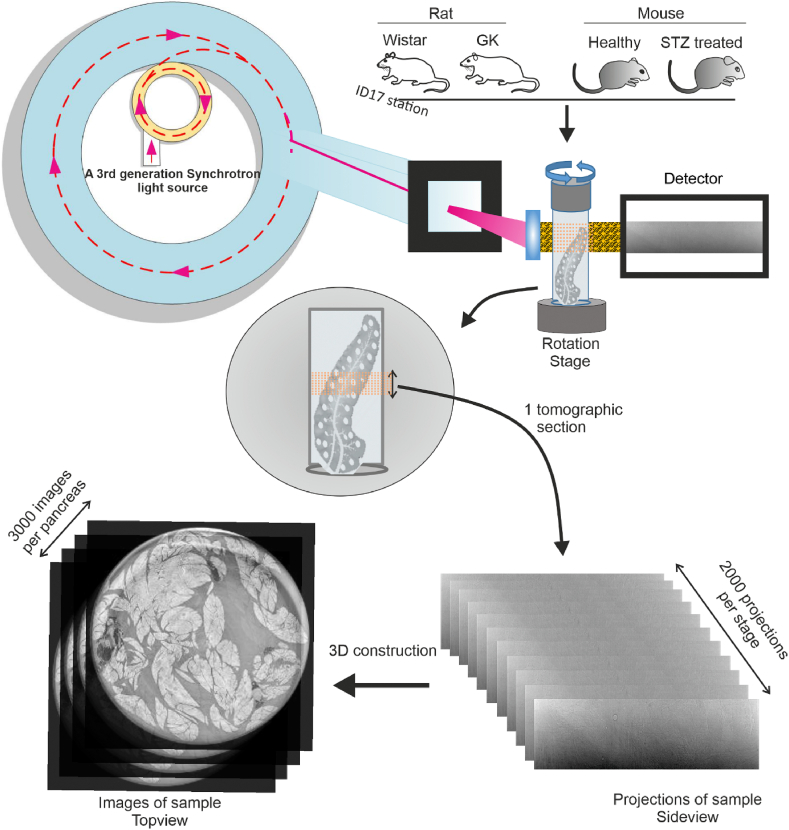
Workflow for pancreas imaging with high-resolution and large-scale size at the biomedical beamline ID17, ESRF. The image acquisition was using the propagation-based X-ray phase-contrast micro-CT, with monochromatic energy of 30 keV. The pancreases were isolated from rats (4 Wistar and 4 diabetic GK) and C57BL mice (4 control and 4 with 5-day STZ treatment). All raw projection images were stored in EDF file format. After the acquisition, the images were reconstructed to visualize the structures of the intact pancreas. Each pancreas sample consisted of 3000 reconstructed images.

A high-resolution and penetrative imaging method was employed to image the entire pancreas based on SRμCT. The microtomography experiments at the ID17-ESRF beamline (Fig. 1) were performed using the monochromatic X-rays with an energy of 30 keV that was ensured to achieve the resolution. A pancreas was imaged by 5–10 tomograms, depending on the height of the organ. For each tomogram, 2000 angular projections were collected, each presenting a size of 3.2 mm (height) × 15.97 mm (width). Following the white field normalization, a phase-retrieval procedure has been applied to the projections to obtain higher contrast. To the end, the structure of a pancreas section was visualize by the 3D tomographic reconstructions (Fig. 1).

### 3.2 The distinct islets structures in the pancreas visualized by SRμCT imaging

**Fig. 2:**
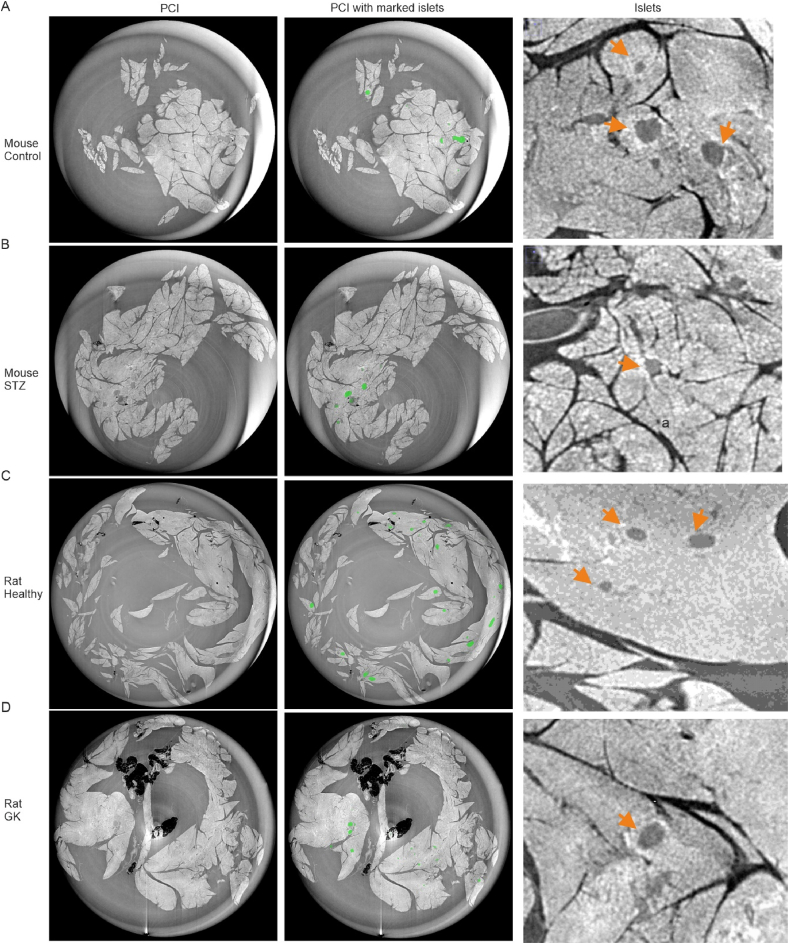
**Representative SRμCT PCI images of the pancreas in healthy and diabetic rodents.** Left, images were acquired and reconstructed with a voxel size of 7.8 × 7.8 × 7.8 μm^3^ at the ID17 beamline. The pancreas images were acquired from healthy mouse (A) STZ treated diabetic mouse (B), healthy Wistar rat (C) and Goto-Kakizaki (GK) diabetic rat (D). Middle, the islets in the pancreas images were marked with green. R**ight**, representative islets in the rodent pancreas. Notice that the number of islets was comparably decreased in the pancreas of both diabetic mice and rats. (For interpretation of the references to color in this figure legend, the reader is referred to the Web version of this article.)

Benefiting the advances of the setup, the significant enhancement in signal-to-noise ratio in the label-free pancreas tissue was achieved. Especially the distinct edge between islets and surrounding tissues enables us to visualize the islets-like structures in the unlabelled tissues (Fig. 2A–D and Fig. S1A). To verify whether the small structures are islets, we have performed immunostaining and identified the islets-like structures by beta cell marker insulin and alpha cell maker glucagon. The confocal imaging confirmed the structures contain a majority insulin-positive cells inside and a minority glucagon-positive cells on the surface (Fig. S1B).

We observed that the number of islets decreased in the diabetic STZ-treated mouse and the GK rat (Fig. 2B and D) compared to the healthy controls (Fig. 2A and C). However, calculating the amounts of all islets in the pancreas is challenging. The conventional methods used to distinguish the islet structures are based on the grey level intensity, the shape or edges of the islets and are unable to distinguish the islet properly (Fig. S2). It is mainly because of the complexity of the imaging conditions and multiple tissue types in the pancreas. We, therefore, pursue to develop a neural network based on the deep learning method to segment the islet.

### 3.3 Segmentation of islets in an entire pancreas by deep learning AA-Net

Compared to other well-established neural network methods, AA-Net prioritizes the reduction of redundant signals resulting from the repeated blocks in the traditional encoder. To obtain richer global contextual information, we developed AAM, an algorithm based on the correlation between the target pixel (blue) and other pixels including long-distance pixels (orange) in the image (Figs. S3A–B). The inserted AAM combined encoder-decoder architecture enhances the feature representation capability and restores the spatial resolution (Figs. S3C and S3D). The result from the penultimate convolution of the decoder is extracted and shown as the 128 × 128 image patch for clarity (Figs. S3E and S4B). To test the performance of AAM, we also applied the AAM with some encoders e.g. Shortcut-Free(SF) blocks or ResNet blocks [22]. Interestingly, each model inserted with AAM performed better than its simplified model without AAM (Fig. S4B and Table S1). Indeed, AAM enables AA-Net retraining full of the advantages in comparison with the methods ResNet or SF block, where the rates of Intersection Over Union (IOU) increased up to 1.47% and 0.68% (Table S1).

**Fig. 3:**
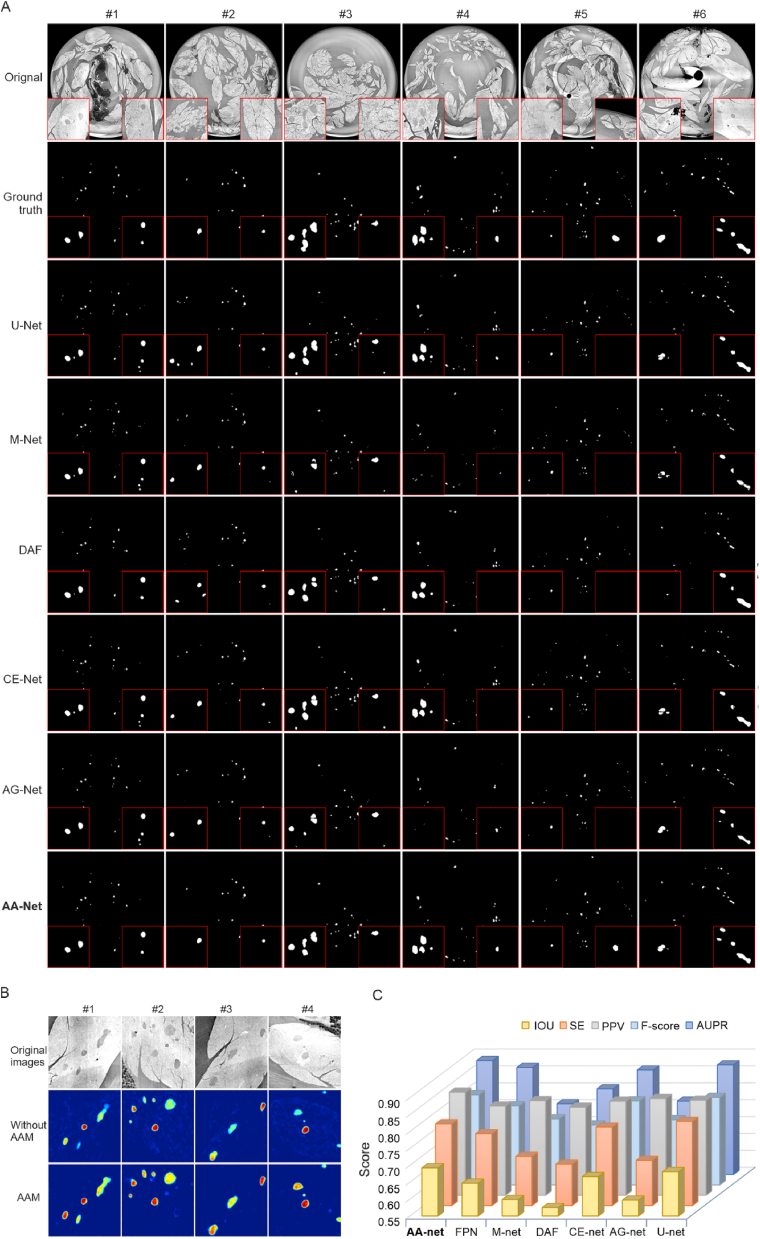
**Comparison of AA-Net with other established deep learning methods.** (A), Representative segmentation results from AA-net and other published methods, including U-Net, M-Net, DAF, CE-Net and AG-Net. The images were randomly selected from the reconstructed image datasets. AA-net ultimately exhibits the advantages for the segmentation, regardless of the size, intensity, or complexity of the background on the pancreas images. (B), Visualization of the islet features in the pancreas by segmentation with or without AAM (Control) in the AA-Net. (C), Statistical comparisons of the islet segmentation among well-established methods, including U-Net, FPN, M-Net, DAF, CE-Net and AG-Net, to AA-Net. The parameters SE, PPV, IOU and AUPR indicate sensitivity, positive predictive value, intersection over union and the area under the precision-recall curve, respectively. F-score was calculated SE and PPV (see the formula in Methods).

Next, we evaluated the effectiveness of AA-Net by comparing it with published state-of-the-art models, including presently widely-used ones in biomedical images processing, e.g., U-Net [26], M-Net [27], DAF [28], CE-Net [29] and AG-Net [30] (Fig. 3). Clearly, the results referring to the ground truth images showed that the segmentation carried out by AA-Net significantly outperformed the existing models. To further validate that the outcome is not due to false-positive targets, we computed the certainty of AA-Net and AG-Net which served as a second of the best performance evaluator (Fig. S5). The data show that the AA-Net achieves a significantly higher certainty rate than AG-Net in the segmentation.

### 3.4 Quantitative analysis of all islets in the whole pancreas

**Fig. 4:**
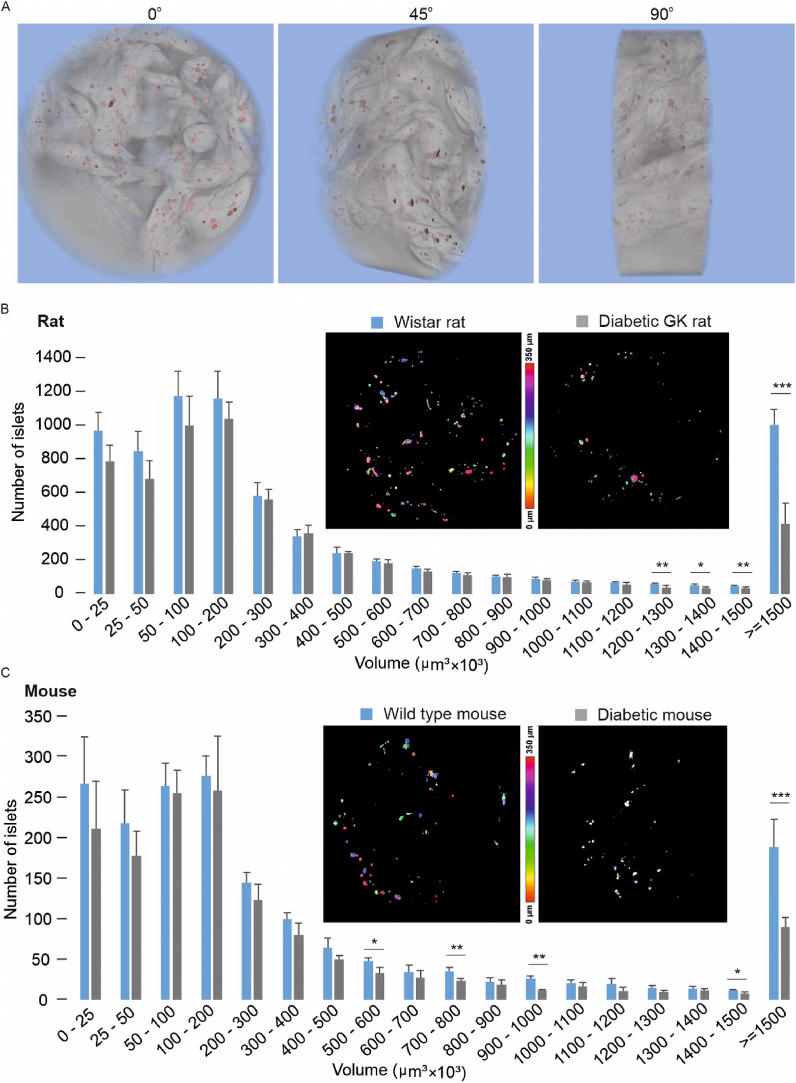
**Quantification of 3D-volume of islets in healthy and diabetic mice and rats.** (A), 3D visualization of a mouse pancreas containing resolved islets. A presentative sideview of the middle-section of the pancreas, as viewed from a viewing angle of 0, 45 and 90°. The reconstructed structures were formed by 500 images collected from the middle of the sample tube. (B), Representative reconstructed images from Wistar and diabetic GK rats (Upper). The volume distribution of the islets in Wistar (n = 4) and GK (n = 4) rats (Bottom). (C), Representative reconstructed images from healthy and STZ-treated mice (Upper). The volume distribution of the islets in C57BL/6 (n = 4) and STZ-treated (n = 4) mice (Bottom). The comparison analysis was performed by a one-way ANOVA test. *p < 0.05, **p < 0.01, **p < 0.001.

We then applied the AA-Net to the reconstructed SRμCT images dataset. The dataset consists of 16 rodent pancreases and each pancreas was made of 3000 reconstructed images (Fig. 4A, Figs. S6A–B). This application allows us to segment all islets from the pancreas and compute their 3D structures. We then calculated the total number of islets in the pancreases. In the wild-type Wistar rat, each pancreas contained 7305 ± 748 islets, while in the diabetic GK rat, the number of islets in the pancreas significantly decreased to 5945 ± 763. In the C57BL/6 mouse, the pancreas contained 1726 ± 201 islets. After destroying the islets with a 5-day streptozotocin (STZ) treatment, the number of islets per pancreas was decreased to 1416 ± 201 (Figs. S6C–D). Though the numbers of quantified islets varied between the two species, it is evident that the diabetic effects caused by either chronic or acute treatment resulted in the reduction of islets.

### 3.5 Decreased volumes of the larger islets in diabetic rodents

After the segmentation and quantification of each islet in the entire pancreas, an extensive population of islets was revealed in the pancreas according to the size distribution from small (25 × 10^3^ μm^3^, ∼8 cells volume) to large (1500 × 10^3^ μm^3^, ∼500 cells volume) (Fig. 4B and C).

We next generated the full distribution of the averaged islets volumes. Intriguingly, the distribution patterns between different animal individuals or species showed no significant differences, confirming that the size of the islets is highly preserved. However, the distribution exhibited a significantly decreased pattern in diabetic conditions, either in chronically developed GK rats (Fig. 4B). or acutely STZ-treated mice (Fig. 4C). From the size distribution, we observed that large islets in acutely STZ-treated diabetic mice are subject to a notable decrease in number >50% (control 189 ± 33 vs diabetes 90 ± 29, n = 4). In diabetic GK rats, the volume reduction occurred predominantly in the large islets. There is no significant difference between diabetes and wild type while the size of the islets was less than 1200 μm^3^ in volume. However, in the large islets (>1500 μm^3^), the size decreased dramatically (wild type 1004 ± 94 vs diabetes 419 ± 122, n = 4) in the GK diabetic rats. These results suggested that the large islets are specifically sensitive to diabetic conditions.

## 4 Discussion

The creation and development of an automated high-resolution imaging approach provides a view of the islet pathological process for both type 1 and type 2 diabetes and further provides clinical indexes for early dialogues of diabetes. In this study, by using SRμCT and phase-contrast imaging, we have created a label-free method for the detection of islets in the pancreas of rodents. Furthermore, we developed the deep learning network AA-Net to segment the scattered islets from the 3D data sets of reconstructed pancreases. The AA-Net showed important advantages, enabling us to identify the destructive damage in the large islets in diabetic rodents.

### 4.1 SRμCT of pancreatic islets

SRμCT in combination with the propagation-based phase contrast imaging (PCI) appeared to be an ideal imaging method for the detection of Langerhans islets in pancreatic tissue. As shown in Fig. 2 and Fig. S1A, the PCI images show a superior contrast between the different features present in the pancreas and the shape of islets has been clearly distinguished from surrounding tissue. The islets-like structure can be verified by laser-based bright light and fluorescence images (Fig. S1B). Additionally, the reduction of islets in the diabetic pancreas was apparent though (Fig. 2), the challenge of islets segmentation and quantification remains. Among the reasons, local intensity values fluctuations due to the presence of image artefacts, different morphology of the islets and the presence of other structures, e.g. blood vessels and exocrine tissues in the images that interfere in the recognition of the structure of interest. Besides, classical methods, based mainly on the object's morphologic differentiation and grey level, cannot achieve automatic islets segmentation (Fig. S2). These results imply the complexity and necessity of segmentation in the analysis of PCI images for the *ex vivo* unlabelled pancreatic islets or other soft tissue. Yet, the success of the PCI in the soft tissue provides the practical supports for further label-free in vivo experiments.

### 4.2 AA-NET and medical image processing

Deep learning-based segmentation methods have recently been applied for medical image analysis. For example, Sinha and Dolz [31] considered the non-local computation method to explore more robust contexts based on the non-local property: the response of one pixel can be affected not only by adjacent pixels, but also by similar pixels far away. This computation lets the spatial distribution of similar objects be utilized through appearance similarity for better global contexts. This work adopts the attention module for the nature-image-oriented model DANet [32] and stakes the attention modules directly. The attention module in DANet [32] also fuses input features and weighted features through addition. In this regard, DANet is unable to increase more channels for features and, therefore, is weak for discrimination. Our AAM, however, takes concatenation for feature fusion so that the discrimination increases with multiple channels (Figs. S3B–D). In addition, integrating AAM in AA-Net can obtain less redundancy than the staked way (Fig. S4A).

The segmentation analysis used by the deep learning method is mainly based on the U-Net style with various encoders [33–38]. DUNet [33] and DRU-Net [34] replace the standard convolutions in the encoder with deformable convolutions, while Gibson et al. [35] introduced dense connection in each encoder block. ResNet block can avoid the gradient vanishing and facilitate the training of deep learning models [39,40]; therefore, ResNet block has been adopted by some methods [[36], [37], [38]]. However, most existing methods overlook the important fact that the encoder may lead to redundant information due to repeated blocks. But the multi-scale feature extraction property of the repeated blocks should be kept even when reducing the redundancy. Therefore, our AA-Net uses a complementary way with a twin-block design so that SF blocks are installed at the beginning and the end of the encoder to partially remove the redundancy while keeping the efficient ResNet block in the middle for avoiding gradient vanishing (Fig. S4A). Compared to only using ResNet block or SF block, AA-Net achieves the best result in metric PPV, AUPR, IOU and F-score (Table S1). Especially, the index of AUPR is significantly higher implicates that the advantage of reduction for error results by using AA-Net.

### 4.3 Quantitative analysis of 3D segmented pancreatic islets in diabetes

Reports in islet transplantation experiments showed that islet volume was significantly reduced in patients with overt hyperglycaemia [41]. In vitro experiments also showed that fluorescence-labelled beta cells [4] are concentrated in diabetic mouse. These reports suggested that diabetic conditions such as glucotoxicity may change islet size. However, no reports extend the comparison to the whole pancreas level. We quantified each islet in the intact pancreas after 3D segmentation of the voxel data, including number and size. We had analysed the pancreas of C57BL/6J mice with control and 5-day STZ treatment which acutely destroys the beta cells in islets. In the STZ-treated mice, the number of islets significantly decreased (from 1766 ± 201 to 1416 ± 230–18%) (Fig. 4C). Likewise, in diabetic GK rats, the number of islets also reduced compared to wild-type rats (from 7305 ± 748 to 5945 ± 763, ∼19%). This significant decrease in islets number reflects the deficiency of insulin secretion carried out by the islets in diabetic rodents.

Report based on fluorescence dye labelling on beta cells has shown that the most common size of islets the most common islet sizes are 32,000–64,000 μm^3^ and in the Type-1 diabetic NOD mice, the all size of islets are reduced [4]. However, we observed that in acutely STZ-treated diabetic mice, the large-size islets are subject to a notable decrease in number >50% (control 189 ± 33 vs diabetes 90 ± 29, n = 4). In diabetic GK rats, the volume reduction occurred predominantly in the large-size islets (Fig. 4B). There is no significant difference between diabetes and wild type, while the islet size was less than 1200 μm^3^ in volume. However, in the large islets (>1500 μm^3^), the size decreased dramatically (wild type 1004 ± 94 vs diabetes 419 ± 122, n = 4) in the GK diabetic rats. Considering that the large size islets are highly sensitive to the diabetic condition, we believe that the large islets should be an important target for clinical application.

Though these findings provide the new observation about the islet volumes alternation, it will be much more crucial if it is so to the in vivo rodents. It is understandable that the in vivo conditions are more complex than the *ex vivo* conditions. Especially, the tissue vibration caused by the breath and blood flow deteriorate the quality of images for example inducing image blur and reducing image resolution. To solve the problems, the development of the new generation of the deep-learning algorithm based on the AA-Net is entirely conceivable. This AA-Net-based method aims to correct and relocate the signal pixel by pixel and improve the errors occurred in in vivo conditions. In addition, it is also proposed to achieve the goal to monitor dynamically the islet volumes following timelines.

In conclusion, this newly established method, combined with SRμCT and PCI, followed by AA-Net analysis, permits an automatic segmentation of unlabelled pancreatic islets, and discerns the changes in islet numbers and volumes in the diabetic pancreas, thus advances the imaging analysis one step toward the preclinical in vivo application. Moreover, this non-destructive method benefits not only for assessing the capacity of insulin secretion and early diagnosis of diabetes but also provides a strategy for soft tissue imaging, such as brain and tumour analysis.

## Author contribution statement

Enming Zhang: Conceived and designed the experiments; Contributed reagents, materials, analysis tools or data; Wrote the paper.

Qingqing Guo; Xiaoping Jiang; Linbo Wang; Emanuel Larsson; Alberto Bravin: Analyzed and interpreted the data.

Abdulla Kazim; Alberto Mittone: Performed the experiments.

Erik Renström: Conceived and designed the experiments; Contributed reagents, materials, analysis tools or data.

Xianyong Fang: Contributed reagents, materials, analysis tools or data; Wrote the paper.

## Funding statement

Dr Enming Zhang was supported by Vetenskapsrådet [2019-01567 and 2018-03258], Diabetes Wellness Network Sverige [1904-PG] and ESRF MD-891.

Xianyong Fang was supported by Natural Science Foundation of Anhui Province [2108085MF210].

## Data availability statement

Data associated with this study has been deposited at https://github.com/AHU-VRV/Deep-Pancreatic-Islets-Segmentaion

## Declaration of interest’s statement

The authors declare no competing interests.
