# Supplementary material for: Reduced volume of diabetic pancreatic islets in rodents detected by synchrotron X-ray phase-contrast microtomography and deep learning network
Source: Heliyon. 2023 Jan 19;9(2):e13081. doi: 10.1016/j.heliyon.2023.e13081 (PMC9883183; doi:10.1016/j.heliyon.2023.e13081)
Supplement: Multimedia component 1 [file mmc1.docx]

**Supplemental information**

**Reduced Volume of Diabetic Pancreatic Islets in Rodents Detected by Synchrotron X-ray Phase-contrast Microtomography and Deep Learning Network**

Qingqing Guo^a, b^, Abdulla AlKendi ^b^, Xiaoping Jiang^b, c^, Alberto Mittone^d, e^, Linbo Wang^a^, Emanuel Larsson^f^, Alberto Bravin^h,i^, Erik Renström^b^, Xianyong Fang^a, *^, Enming Zhang^b, g, *^

a, School of Computer Science and Technology, Anhui University, Hefei, China.

b, Islet Pathophysiology, Department of Clinical Science, Lund University Diabetes Centre, Malmö, Sweden.

c, School of Physical Science and Technology, Southwest University, Chongqing, China.

d, Advanced Photon Source, Argonne National Laboratory, Lemont, IL, United States.

e Biomedical Beamline ID17, European Synchrotron Radiation Facility, Grenoble Cedex, France.

f, Division of Solid Mechanics & LUNARC, Department of Construction Sciences, Lund University, Lund, Sweden.

g, NanoLund, Lund University, Box 118, 22100, Lund, Sweden.

h, Department of Physics, University Milano Bicocca, Milan, Italy

i, Department of Physics, Università della Calabria, Rende, Italy

*, Correspondence should be addressed to X. F. (fangxianyong@ahu.edu.cn) or E. Z. (enming.zhang@med.lu.se)

Keywords: Diabetes; Pancreatic islets; X-ray microtomography; Deep learning; Synchrotron radiation

The Supplementary information includes Table S1 and Figs. S1 to S6

**Table S1, The parameters indicate the effectiveness and accuracy of segmentation with AAM** (More explanation in Methods). ‘SF only’ and ‘ResNet only’ represent the simplified AA-Net with only the SF and ResNet blocks in the encoder, respectively, while ‘Yes’ or ‘No’ indicate whether AAM is incorporated into the models. The best results are shown in bold. All metric are computed at randomly selected images(n = 19). The comparison analysis was performed by a student T-test. * p < 0.05. Please note that the index of AUPR is significantly enhanced under conditions with AAM-incorporated AA-Net.

| **Model** | **AAM** | **SE** | **PPV** | **AUPR** | **IOU** | **F-score** |
| --- | --- | --- | --- | --- | --- | --- |
| **SF only** | **No** | **0.7993±0.0755** | 0.8313±0.0901 | 0.8746±0.0445 | 0.6813±0.0649 | 0.8088±0.0469 |
|  | **Yes** | 0.7996±0.0736 | **0.8405±0.0768** | **0.8827±0.0335** | **0.6886±0.0502** | **0.8144±0.0383** |
| **ResNet only** | **No** | 0.7845±0.0821 | 0.8326±0.0899 | 0.8691±0.0523 | 0.6734±0.0769 | 0.8024±0.0556 |
|  | **Yes** | **0.7861±0.0741** | **0.8358±0.0845** | **0.8724±0.0500** | **0.6780±0.0738** | **0.8059±0.0527** |
| **AA-Net** | **No** | **0.7927±0.0779** | 0.8465±0.8404 | 0.8764±0.0513 | 0.6881±0.0678 | 0.8135±0.0476 |
|  | **Yes** | 0.7922±0.0725 | **0.8551±0.0873** | **0.8873±0.0418**^*^ | **0.6924±0.0626** | **0.8168±0.0434** |


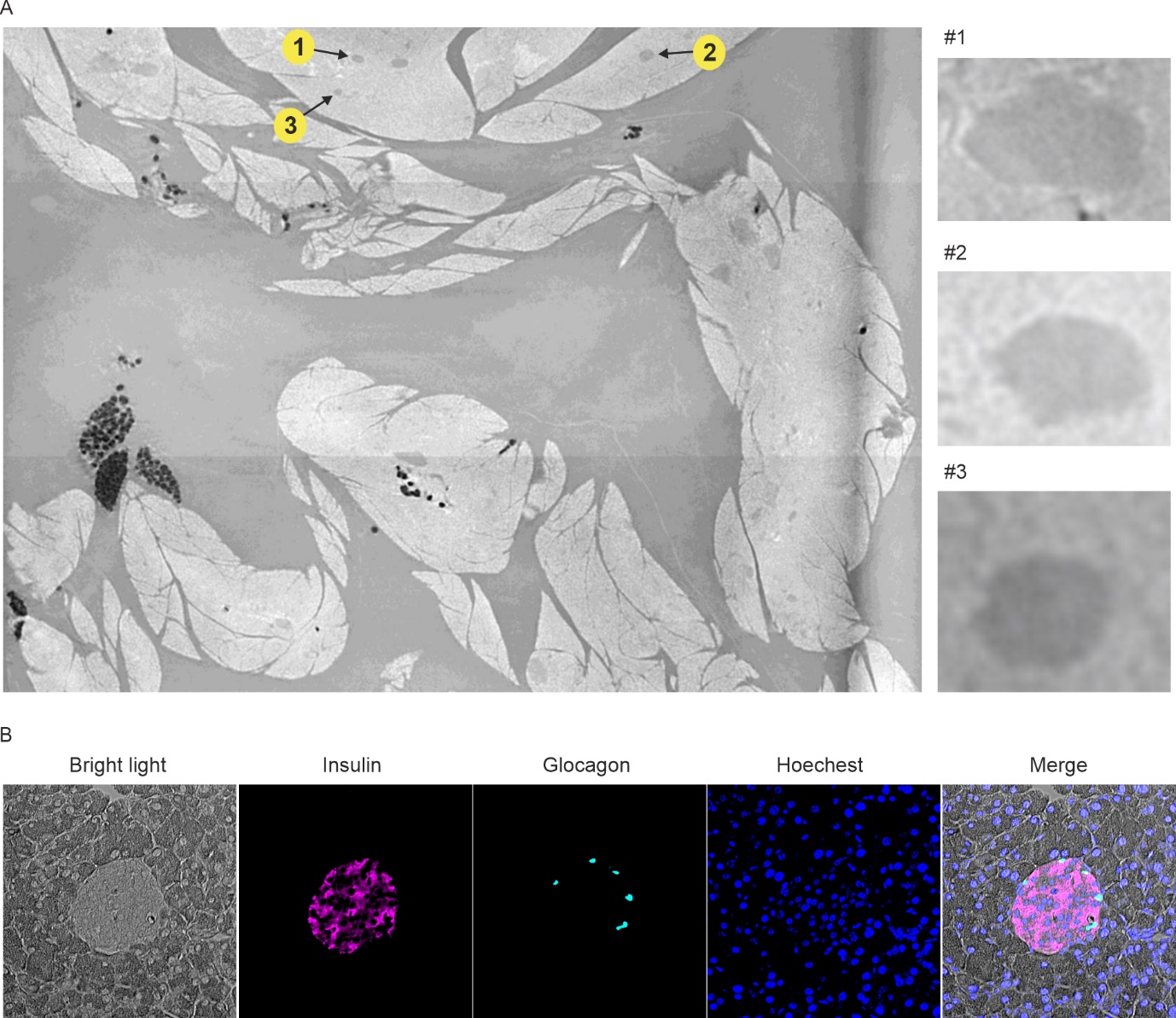


Fig. S1 Islets structures were visualized by the Synchrotron X-ray phase-contrast microtomography. (A) Three representative islets have been highlighted in a reconstructed slice of the pancreas. The islets exhibited clear differences including intensity, shapes and edges distinguished from neighboring tissue. **(B**) Confocal imaging of the section of the pancreas slide (5-µm thickness) to confirm the islet structure with immunostaining. The islet structures were obtained by normal transmitted light. Their main contained cells, i.e. beta cells, alpha cells and other types of cells, were labelled by insulin, glucagon and nuclear dye Hoechst, respectively.


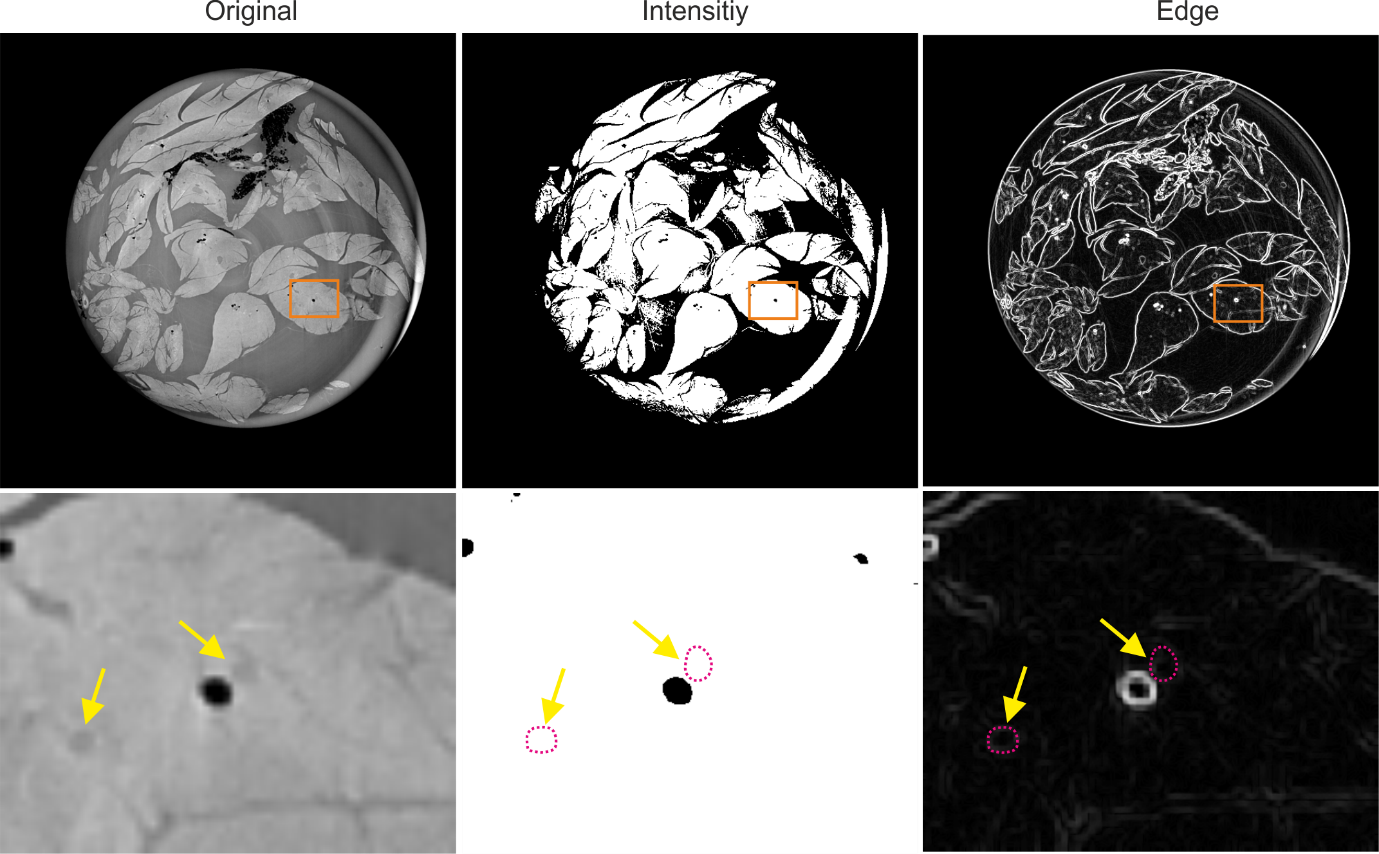


Fig. S2 The islet structures cannot be segmented by conventional segmentation methods. Segmentation of islet structures used conventional methods based on intensity or edge contrast. Unexpectedly, the islets are unable to be segmented by either grey-level-based methods due to their low grey level intensities or by edge-based methods due to the high-contrast edges in neighboring tissues. Note: in sub-figures, the brightness and contrast have been adjusted to sufficiently illustrate the features inside the slices.


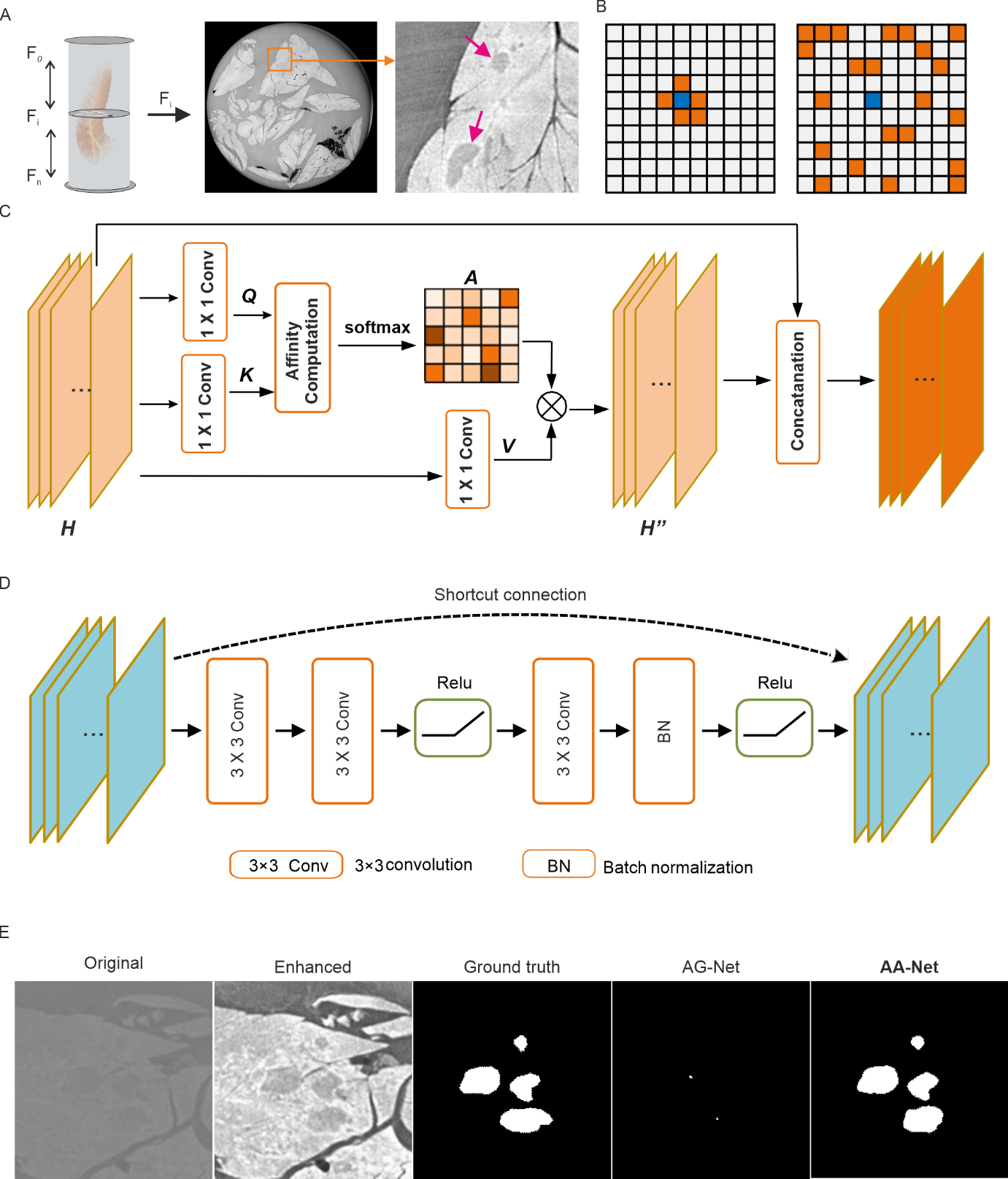


Fig. S3 **The deep learning network, AA-Net, incorporated with AAM were created for the islet segmentation.** **(A)**, Representative phase-contrast slice from the middle section of the pancreas. Note that the arrows indicate islet structures from a zoom-ed in the region. **(B)**, Principle of AAM with a blue pixel from an islet and similar ones in yellow. AAM relies on the globally distributed similar pixels (right) that differ from the established methods mainly depending on locally similar pixels (left). **(C)**, Structure of AAM. An affinity measurement between feature Q and K is estimated by the Affinity Computation. The matrix multiplication is performed flattened V and A to obtain a weighted feature H′′. **(D)**, Comparison of the designs between the ResNet and SF blocks. The dashed shortcut connection appears in the ResNet block, which does not exist in the SF block. (E) An example islet image and segmentation results from AA-Net, ground truth and published AG-Net. “Enhanced” indicated the image was proceeded to enhance the brightness and contrast.


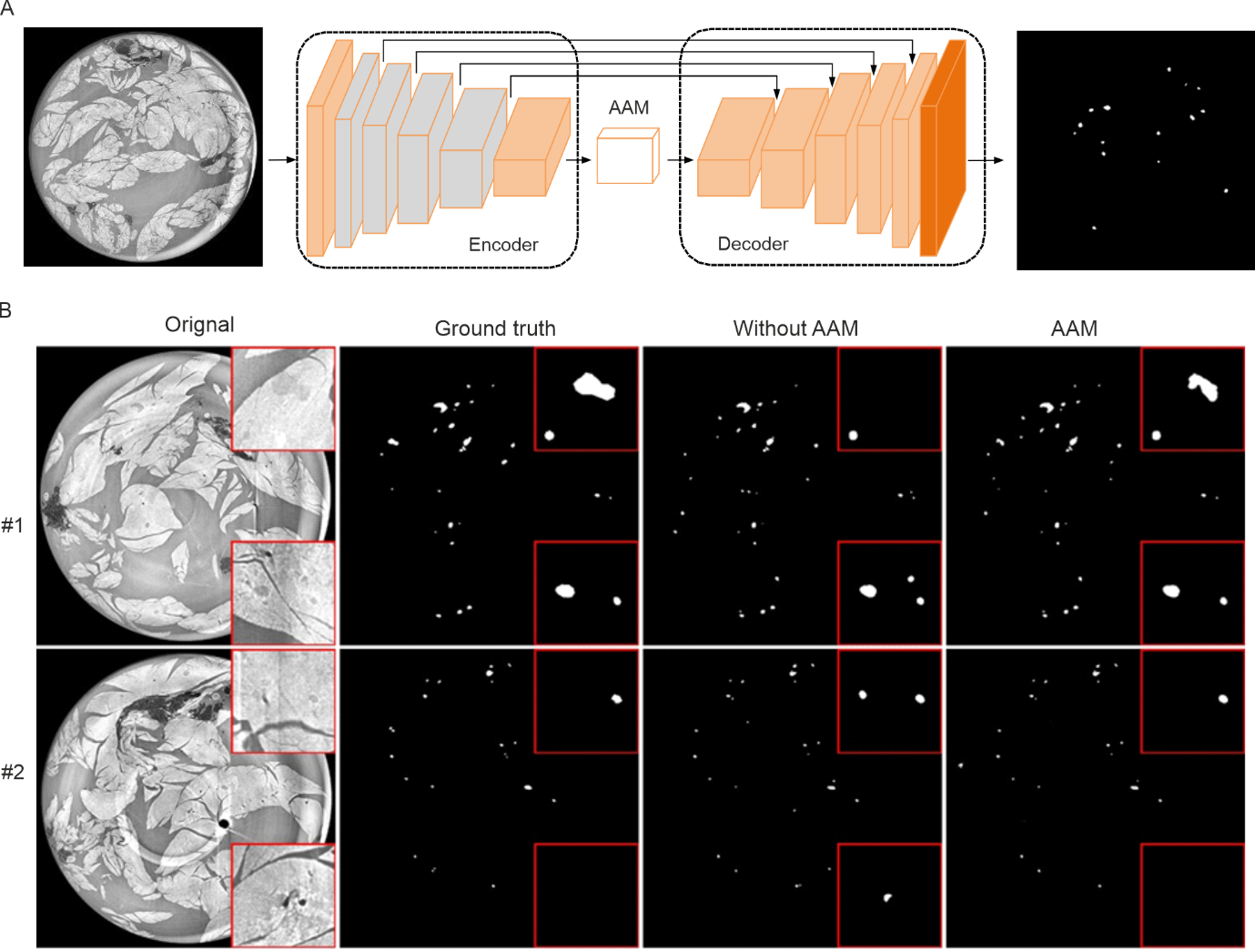


Fig. S4 **Ablation analysis of AAM and the twin-block encoder of AA-Net. (A)**, The AA-Net structure consists of three parts, encoder, AAM, and decoder modules. **(B)**, Representative images show the performance comparisons of AA-Net with or without AAM.


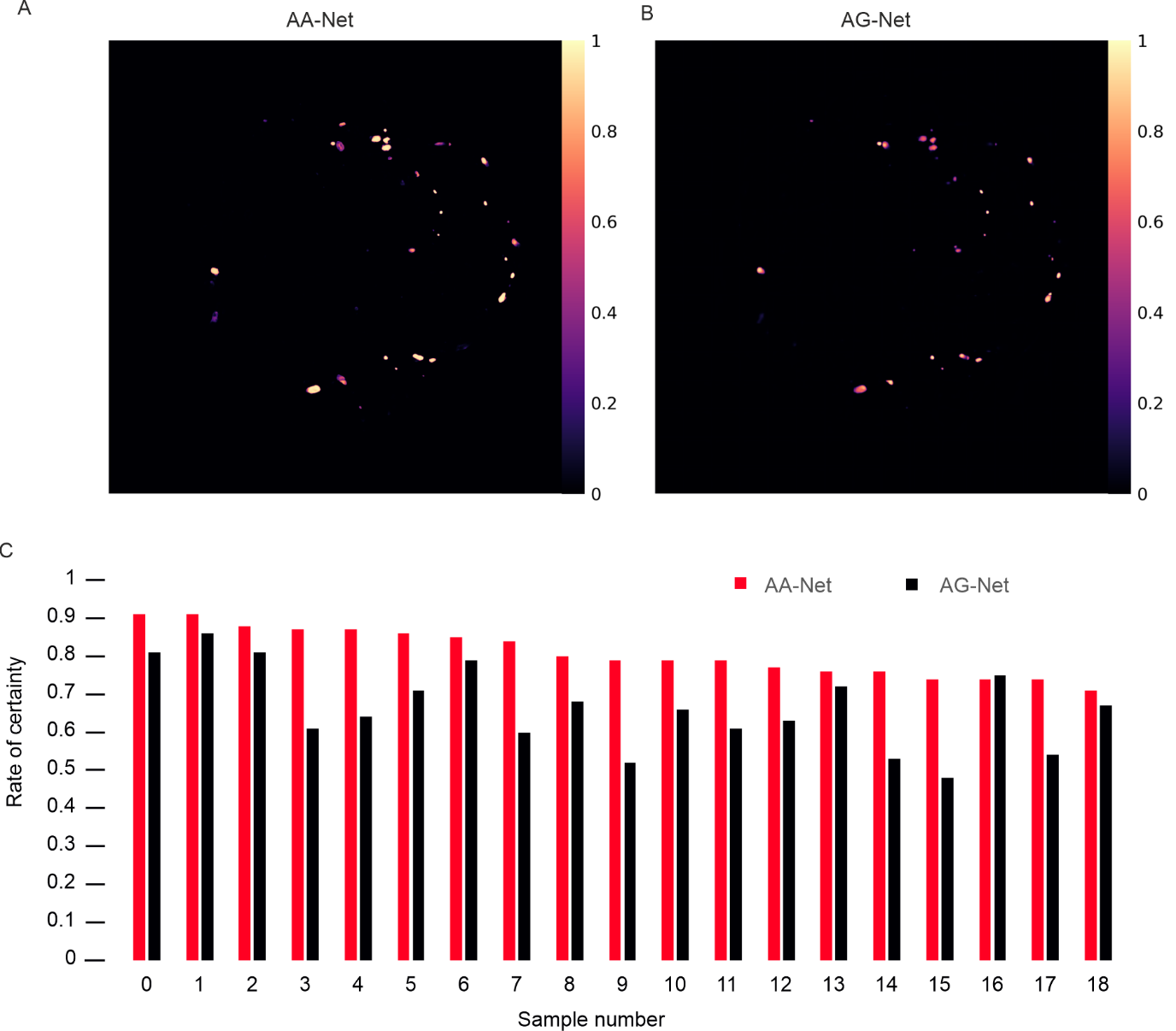


Fig. S5 **High certainty rates of the islets segmentation were achieved by AA-Net analysis.**  (A**)** Visualization of the certainty map from the uncertainty analysis of AA-Net. The scale of 0 to 1 indicates the certainty rate from uncertainty to complete certainty. (**B)** same as in (**A)**, but performed by published AG-net. (**C**) Quantitative analysis of the certainty from randomly selected samples of pancreas images. Note that AA-net has higher rates of certainty in comparison with established AG-Net.


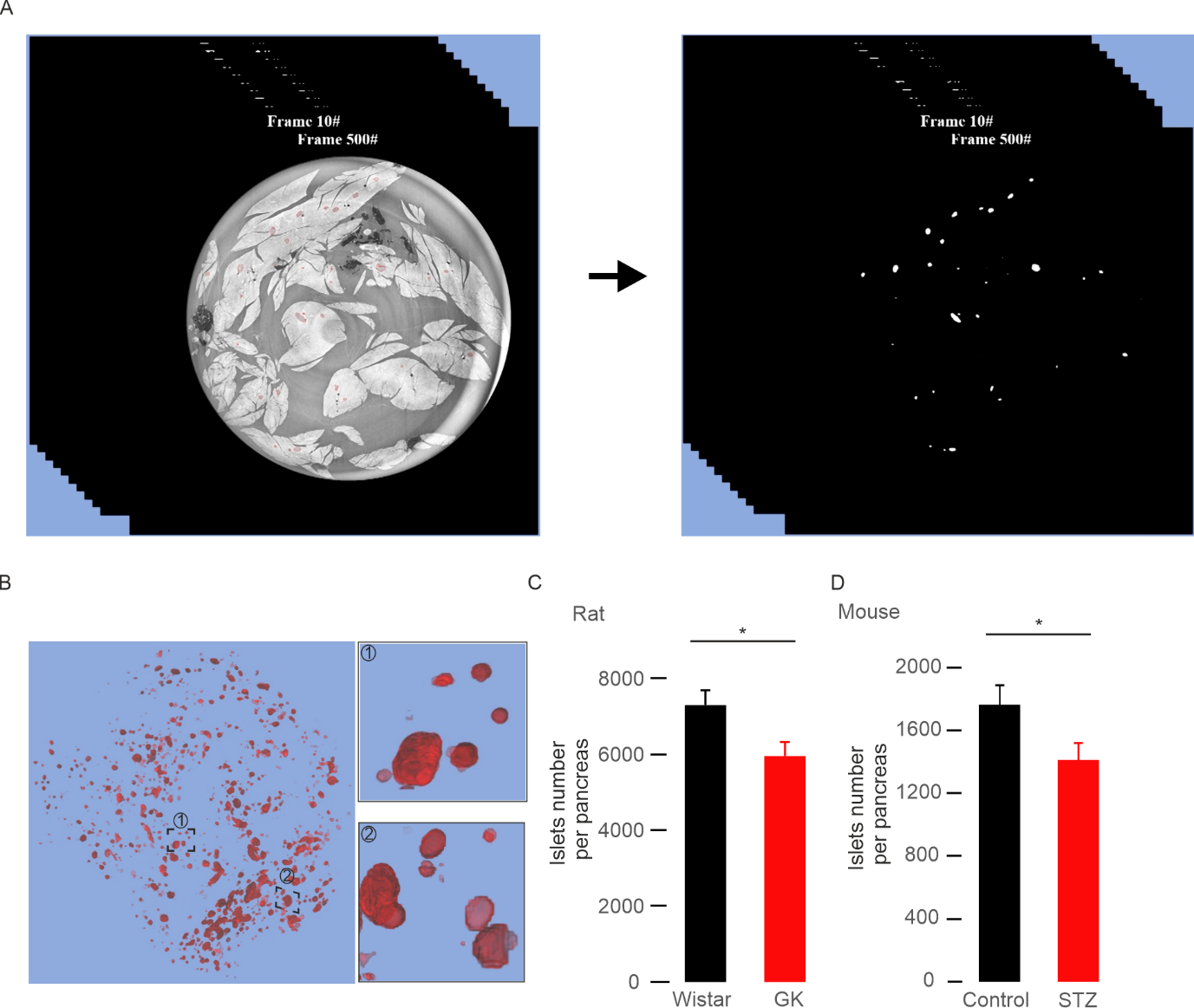


Fig. S6 3D segmentation of islets from the intact pancreas images. A) Segmentation of islets from 500 pancreas slices using AA-net. Note that the edge of islets is drawn by a red line. **B)**, Representative result of 3D segmented islets from the middle section of the pancreas. Zoomed islets from the reconstructed images with the front view (1) and 45-degree side view (2). C), the total number of islets in the pancreas computed on the segmented slices of the islets in healthy (n=4) and diabetic GK (n=4) rats. **(D)**, Same as in **c**, but in C57BL mice with control (n=4) or 5-day STZ (200 mg/kg) treatment (n=4). The data is represented by Mean±SEM. The comparison analysis was performed by the two-tailed Student’s T-test. * p < 0.05.
